# Time-Varying Associations Between Device-Based and Ecological Momentary Assessment–Reported Sedentary Behaviors and the Concurrent Affective States Among Adolescents: Proof-of-Concept Study

**DOI:** 10.2196/37743

**Published:** 2022-06-10

**Authors:** Jennifer Zink, Chih-Hsiang Yang, Jasmin M Alves, Kelsey L McAlister, Jimi Huh, Mary Ann Pentz, Kathleen A Page, Genevieve F Dunton, Britni R Belcher

**Affiliations:** 1 Department of Population and Public Health Sciences Keck School of Medicine University of Southern California Los Angeles, CA United States; 2 Department of Exercise Science Arnold School of Public Health University of South Carolina Columbia, SC United States; 3 Department of Medicine Keck School of Medicine University of Southern California Los Angeles, CA United States; 4 Department of Psychology University of Southern California Los Angeles, CA United States

**Keywords:** accelerometry, intensive longitudinal data, mood, youth, mobile phone

## Abstract

**Background:**

Previous studies on affective state–sedentary behavior (SB) associations have not accounted for their potentially time-varying nature and have used inconsistent SB measurement modalities. We investigated whether the strength of the associations between affective states and SB varied as a function of the time of day and by SB measurement modality (device-measured SB vs ecological momentary assessment–reported screen-based SB) in youth.

**Objective:**

This study aimed to establish a proof of concept that SB–affective state associations may not be static during the day. In addition, we aimed to inform the methodology of future work, which may need to model associations as functions of the time of day and carefully consider how SB is operationalized or measured.

**Methods:**

A total of 15 adolescents (age: mean 13.07, SD 1.03 years; 10/15, 67% female; 6/15, 40% Hispanic; 10/15, 67% healthy weight) wore thigh-mounted activPAL accelerometers and simultaneously reported their screen-based SBs and concurrent positive and negative affective states via ecological momentary assessment for 7 to 14 days (N=636 occasions). Time-varying effect models (varying slopes) examined how each measure of SB was associated with concurrent affective states from 7 AM to 8 PM.

**Results:**

Time-varying effect model plots revealed that these associations varied in strength throughout the day. Specifically, device-based SB was related to greater concurrent negative affect only after approximately 5 PM and was unrelated to concurrent positive affect. Screen-based SB was related to greater concurrent negative affect only from 7 AM to approximately 9 AM. This was also related to greater concurrent positive affect from 7 AM to approximately 9:30 AM and from approximately 3 PM to approximately 7 PM.

**Conclusions:**

We provide preliminary evidence to suggest that future confirmatory studies investigating the SB–affective state relationship should consider the time-varying nature of these associations and SB measurement modality. There may be critical time windows when specific types of SBs co-occur with affect, suggesting that interventions may need tailoring to the time of day and type of SB if future studies using similar methodologies can replicate our findings.

## Introduction

### Background

Sedentary behaviors (SBs), such as screen-based behaviors, are highly prevalent among adolescents and are associated with poor health outcomes [1]. Over 30% and 40% of adolescents in the United States report ≥3 hours of daily television viewing and computer use, respectively [2]. The pervasiveness of SB among young people may be attributed to factors such as urbanization [3], technological advancements [4], and the development of social media [5]. Therefore, opportunities for being sedentary will continue to surround youth. Interventions aimed at reducing SB have thus far been relatively ineffective [6]. Understanding the potential correlates of SBs as they occur naturally in everyday life may be important for the development of effective intervention strategies aimed at reducing sedentary time.

Affective states may be an important factor to consider in future behavior change interventions. Acutely, positive affective states can be related to salubrious behaviors such as physical activity [7-9], whereas negative affective states are associated with unfavorable behaviors such as fast-food consumption [10] and cigarette smoking [11] among youth. Therefore, it is plausible that affective states and SB may co-occur during adolescence, a developmental period when sedentariness increases, and affect can become less positive and more variable [12,13]. However, extant studies on the association between affective states and SB among youth have yielded inconsistent findings [8,14,15].

Inconsistencies in the literature thus far may be attributed to unaccounted-for complexities in the association between affective states and SBs, such as their potentially time-varying nature. For example, evidence suggests that SB specifically in the evening may be linked to emotional health outcomes, including worse affect [16,17]. Ecological momentary assessment (EMA) is a data collection method whereby participants report their affective states and behaviors via mobile devices as they occur naturally in real time. It is an ideal method for capturing acute changes in affective states, SBs, and their associations with one another across the day [18]. The time-varying effect model (TVEM) is also being increasingly used in the field of behavioral medicine, especially for leveraging EMA data [19,20]. The TVEM optimizes the repeated-measures data structure of EMA to model dynamic associations over time (across the day). This allows investigators to pinpoint the specific time windows in which associations may be strongest or weakest between 2 variables [21,22]. To our knowledge, previous investigations of the within-day associations between affective states and SB have yet to simultaneously use EMA and the TVEM to assess whether the strength of these associations varies across the chronological time of day. Therefore, our understanding of the potentially time-specific co-occurrence of affective states and SB is limited.

We also have a limited understanding of the potential associations at hand because previous studies have used inconsistent measures or operationalizations of SB. Studies of device-based SB (capturing time spent sitting or lying down) and affective states among youth indicate that negative affect is directly related to SB, whereas positive affect is inversely related to SB [8,14]. In contrast, another study on the associations between EMA-reported screen-based SB (capturing behaviors done while sitting or lying) and affective states among youth yielded null findings [15]. It is unclear whether these conflicting findings are because of measurement and recall errors associated with subjective measures of behavior or whether device-based and subjective SB represent distinct constructs that are differentially related to affective states. Thus, studies with more rigorous approaches, such as those that combine device-based and EMA-reported SB, are needed to elucidate the possible associations at hand.

### Objectives

Taken together, the next step in this area of research is to examine the potential differences in the strength of the within-day associations between affective states and SB by the time of day and operationalization of SB. Therefore, our goal was to provide a proof-of-concept study that would inform the methodology of future work in the topic area of affective states and SB, which may need to account for the time-varying nature of these associations and carefully consider how SB is operationalized moving forward. On the basis of the abovementioned work [8,14,16,17], we hypothesized that higher negative affect, lower positive affect, and SB would co-occur in the evening hours, specifically for device-based SB. The findings of this study can increase our understanding of the relationships at hand by (1) identifying the specific time points within the day that these associations are most likely to occur and (2) identifying which operationalization or measure of SB relates to affective states the most strongly.

## Methods

### Participants and Recruitment

The participants (N=15) in this study were a subset of participants from the Mothers’ and Their Children’s Health (MATCH) cohort study of maternal stress and their children’s obesity risk, which took place in the broader Los Angeles, California, United States, metropolitan area [23]. MATCH study participants were recruited via flyers and in-person research staff visits to public elementary schools and community events. The inclusion criteria for mother-child dyads in the MATCH study were as follows: (1) the child is in third to sixth grade at baseline, (2) more than half of the child’s custody belongs to the mother, and (3) both mother and child can read English or Spanish. Dyads were excluded from the MATCH cohort if the mother or the child (1) was taking medications for thyroid function or psychological conditions, (2) had a health condition that limited physical activity, (3) was enrolled in a special education program, (4) was currently using oral or inhalant corticosteroids for asthma, (5) was pregnant, (6) was classified as underweight by a BMI percentile of <5% adjusted for sex and age (child only), or (7) worked >2 weekday evenings (between 5 and 9 PM) per week or >8 hours on any weekend day (mother only). The detailed MATCH study protocol can be found elsewhere [23].

Participants enrolled in the MATCH study were recruited for the Sedentary Behavior and Health Outcomes Study, an in-laboratory randomized crossover trial investigating the metabolic effects of interrupting sitting (ClinicalTrials.gov NCT03153930). To be eligible for the Sedentary Behavior and Health Outcomes Study (and therefore this substudy), participants were required to be enrolled in the MATCH cohort across all 6 waves (3 years) and be in good general health. Youth with cardiac or pulmonary disease, allergies to metals, evidence of type 2 diabetes, and endocrinologic disorders leading to obesity or taking medications for attention-deficit/hyperactivity disorder were excluded.

### Procedures

Data collection for this study was conducted from May to November 2018. The work presented here is a secondary analysis of pilot data with the primary goal of demonstrating the scalability of combining EMA and continuous glucose monitoring [24]. Enrollment for this pilot study was performed on a rolling basis until the target sample size (N=15) was met. At the baseline screening visit, the participants and their parents provided assent and consent, respectively. Participants reported demographic characteristics, including age, sex, ethnicity, and highest maternal education achieved, which was used as a proxy for socioeconomic status. Anthropometric measurements (height in centimeters and weight in kilograms) were collected in duplicate by trained study staff and used to calculate the age-adjusted BMI percentile using the Centers for Disease Control and Prevention *EpiInfo* tool.

Eligible participants were then instructed to wear an activPAL micro4 accelerometer (PAL Technologies) on their right thigh for 24 hours per day for the next 7 complete days. They were instructed to wear the device at all times, including on school and summer camp days, during extracurricular activities, and during showering or bathing. The study staff placed a waterproof cover on the activPAL and taped the devices to the mid–right thigh to ensure proper placement and minimize participant removal of the device during the assessment period. ActivPALs have been validated for use with youth, and as they are thigh mounted, they can differentiate between sitting and standing, making them ideal for capturing SB [25].

Participants were also provided a Moto G mobile phone (Motorola Mobility) with the *Movisens* EMA app predownloaded for the duration of the study. On weekend days, each participant received 7 random EMA prompts during specified 1-hour time windows between 7 AM and 8 PM. On weekdays, participants received 4 random prompts during specified 1-hour time windows between 7 AM and 8 PM (except during school or summer camp hours, defined as weekdays from 8 AM to 3 PM). Therefore, participants received up to 34 EMA prompts (n=14, 41% on weekend days and n=20, 59% on weekdays) across the 7-day assessment period. The 1-hour EMA prompt time windows are listed in Multimedia Appendix 1. The EMA prompts occurred during the same 7 days when the participants wore the activPAL device. The EMA mobile device chimed and vibrated to prompt the participant to stop their current activity and answer the EMA survey, which took approximately 2 minutes to complete. If a participant did not respond to the EMA survey after the initial alert, there were up to 5 reminder signals within 20 minutes of the initial alert. The EMA survey expired after the fifth alert was ignored. At each prompt, participants were asked to report on their concurrent SBs and affective states. All EMA survey responses were date- and time-stamped.

As part of the Sedentary Behavior and Health Outcomes Study, after the initial 7-day assessment period described previously, participants were asked to return the activPALs and mobile devices to the study team and complete the same 7-day study protocol again (after a washout period ranging from 1 week to approximately 1 month). Therefore, all 15 participants had the opportunity to contribute 14 assessment days (2 separate 7-day observational periods) to the data in this study. Of the 15 participants, 14 (93%) contributed 14 assessment days of data and 1 (7%) participant contributed 7 assessment days of data, all of which were included in the present analyses. Depending on the randomization order from the Sedentary Behavior and Health Outcomes Study in-laboratory randomized trial, all participants were additionally given a wrist-worn activity monitor (LYCOS Life). This device was programmed to prompt participants to interrupt their SB (eg, with walking) every 30 minutes using vibrations for either the first or second assessment week. During the assessment periods when the wrist-worn activity monitor was not assigned to the participants, the study staff instructed the participants to proceed with their normal daily routines. Of the 15 participants, 7 (47%) received LYCOS Life during the first assessment week and 8 (53%) received LYCOS Life during the second observational week. Randomization order (whether the participant received LYCOS Life during the first or second assessment week) did not differ by participant characteristics (age: *P*=.80; sex: *P*=.71; highest maternal education: *P*=.18; ethnicity: *P*=.83; weight status: *P*=.71).

### Measures

#### Device-Based SB

All sleep and nonwear times were removed before analyzing activPAL-measured SB. Nonwear was defined as ≥60 consecutive minutes of 0 counts [26], whereas valid days were defined as ≥10 hours of valid wear time during waking hours [27]. SB was defined as activities requiring ≤1.5 metabolic equivalents [28], and the total number of minutes spent in SB was calculated for the matched 15-minute time window before the EMA prompt. This 15-minute time frame is consistent with previous studies of affective states and SB among youth [19,29] and was considered the smallest meaningful amount of time that matched the presented EMA screen-based SB item wording (eg, *just before the phone went off*).

#### Self-reported Screen-Based SB

Via EMA, participants were asked to select the primary SB that they were currently engaged in at the time of the EMA prompt (*just before the phone went off*). The response options were television, movies, or videos; social media (Facebook, Snapchat, Instagram, and Tumblr); videogames; computer or tablet use; homework or reading; hanging out or chatting; art, painting, or coloring; riding in the car or bus; and none of these items. This item was dummy coded into 1 dichotomous screen-based SB variable, where 1=*yes* to screen-based SB (television, movies, or videos; social media; videogames; and computer or tablet use) versus 0=*no* to screen-based SB (homework/reading; hanging out or chatting; art, painting, or coloring; riding in the car or bus; and none of these things).

#### Affective States

EMA questions prompted participants to report on their current affective states (*just before the phone went off*) based on five items of the Positive and Negative Affect Schedule–Child short form—stressed, mad, sad, happy, and joyful—consistent with previous EMA studies of affective states among youth [10,19]. The response options ranged from 0 to 3 (0*=not at all*, 1*=a little*, 2*=quite a bit*, and 3*=extremely*). The responses for stressed, mad, and sad (3 items) were averaged to create a continuous score for negative affect (within-subject internal consistency reliability, ω=0.81), and the responses for happy and joyful (2 items) were averaged to create a continuous score for positive affect (ω=0.90). Therefore, negative and positive affect could each range from 0 to 3 at any given EMA prompt, with higher scores indicating higher negative or positive affect.

#### Covariates

Time-invariant covariates were selected a priori based on previous work showing associations between SB and symptoms of emotional disorders, including age (continuous; years), sex (dichotomous; female, 1=yes vs 0=no), ethnicity (dichotomous; Hispanic, 1=yes vs 0=no), socioeconomic status (dichotomous; maternal education college or higher, 1=yes vs 0=no), and weight status (based on BMI percentile; dichotomous; overweight/obese, 1=yes vs 0=no) [30-32]. In addition, the time-varying covariate, day of the week (dichotomous; weekend, 1=yes vs 0=no), was included in all models. EMA-reported physical activity (dichotomous; any physical activity, 1=yes vs 0=no), environmental context (dichotomous; indoors, 1=yes vs 0=no), social context (dichotomous; alone, 1=yes vs 0=no), and experimental condition (LYCOS Life Band, 1=yes vs 0=no) were each tested one at a time as covariates and were retained in the models if they were significantly associated with the outcome at the *P*<.05 level. Multimedia Appendix 2 provides a more detailed description of each EMA item.

### Statistical Analysis

Frequencies or means were calculated for participant characteristics, affective states, activPAL-measured SB (in the 15-minute time window before the EMA prompt), and EMA-reported screen-based SB (*yes* to any screen-based SB *just before the phone went off*). Cross-tabulations were used to calculate the mean affective state score based on yes or no reports of screen-based SB. Sample- and individual-level EMA prompt compliances were calculated as the proportion of prompts completed out of the total number of prompts sent to the participants. To better understand the potential patterns of data missingness, separate multilevel logistic regression models tested whether participant age, sex, ethnicity, weight status (healthy weight vs overweight or obese), maternal education, day of the week, week-level positive affect, and week-level negative affected predicted momentary EMA prompt compliance (prompt completed; yes vs no) and valid activPAL wear (valid day; yes vs no). Multimedia Appendix 3 presents the descriptive statistics of EMA prompt compliance.

TVEMs, which are uniquely suited for the analysis of intensive, repeated measures (eg, time-stamped activPAL and EMA data), were used to model the associations between affective states and SB across the day. TVEMs are designed to test changes in the strength of the association between the predictor and outcome over time, which is modeled nonparametrically. Moreover, TVEMs can accommodate an unequal temporal spacing of observations and an unequal number of observations per participant because of missing observations, which is common in EMA studies [22]. The TVEM results are presented graphically, where time is on the x-axis. From a single TVEM, 2 graphical results were produced: (1) an intercept function, which represents momentary levels of affect (the outcome) for participants with average levels of all covariates at a given time, and (2) a slope function, which represents the adjusted estimate of the association (*β*) between SB and concurrent affect at any given time. In this study, the solid line in the figures representing the graphical results represents the point estimate, whereas the dashed lines represent the corresponding 95% CIs. A CI (dashed lines) that does not overlap with zero at any moment in time for the slope function indicates a significant association between the predictor (SB) and outcome (affective states) during a specific time interval. All models in this study presented results from 7 AM to 8 PM because of the EMA sampling protocol.

TVEMs were conducted using %TVEM SAS macro [21]. The TVEMs were fitted using the default setting that applied the penalized truncated power spline (P-spline) technique with 10 knots (dividing points) for computational flexibility and efficiency [19]. In contrast to the unpenalized (B-spline) technique, the P-spline method uses an automated model selection procedure, making it the preferred method for fitting TVEMs that can have complex coefficient functions [21,22]. Therefore, the P-spline approach is more appropriate for modeling momentary within-day changes captured in EMA studies on health behavior [33].

Two empty TVEMs (with only an intercept function and an error term as predictors) were used to describe the unadjusted average levels of (1) negative affect and (2) positive affect reported across the day. To model the associations of interest, four conditional TVEMs were used to assess the changes in the strength of the association between (1) activPAL-measured SB and concurrent negative affect across the day, (2) EMA-reported screen-based SB and concurrent negative affect across the day, (3) activPAL-measured SB and concurrent positive affect across the day, and (4) EMA-reported screen-based SB and concurrent positive affect across the day.

As one of our exposure variables of interest (screen-based SB) encompassed smartphone use, sensitivity analyses were conducted by removing observations from participants (2/15, 13%) who had no prior smartphone ownership. This was performed to assess how providing a study smartphone to those who may otherwise not have had regular access to a smartphone could have influenced our study findings. To understand how the day of the week may have influenced our results, additional sensitivity analyses were conducted to examine the acute associations on weekend days only. Furthermore, as evidence suggests that engagement in screen-based SB may differ on weekend days versus weekdays among youth [34], we calculated frequencies of the reported screen-based SB stratified by the time of day and day of the week (weekend day vs weekday) to investigate whether this was observed in our sample. All analyses were conducted using SAS (version 9.4).

### Ethics Approval

All study procedures were approved by the University of Southern California Institutional Review Board (HS-17-00126).

## Results

### ActivPAL and EMA Compliance

Table 1 shows the characteristics of the study sample. Of the 1030 EMA prompts received, participants completed 636 (61.74% sample-level compliance); thus, the analytic sample size was 636 for TVEMs that leveraged only EMA items. Participant-level EMA compliance ranged from 32.47% to 88%. The multilevel logistic regression analyses of momentary prompt-level EMA compliance indicated that participant characteristics (age, sex, ethnicity, maternal education, weight status, person mean negative affect, and person mean positive affect) were unrelated to EMA prompt compliance (all *P*>.18). The day of the week was also unrelated to momentary EMA prompt compliance (*P*=.39). Participants were more likely to complete EMA prompts later in the day (odds ratio 1.08, 95% CI 1.01-1.14; *P*=.02).

Of the 636 completed EMA prompts, 94 (14.8%) were removed as they were paired with activPAL observations that occurred on nonvalid days. This yielded an analytic sample size of 542 activPAL-matched EMA prompts for TVEMs, where device-based SB (in the past 15 minutes) was the predictor of affective states. The multilevel logistic regression analyses of valid activPAL wear at the day level indicated that participant characteristics, including person mean affective states, were unrelated to valid wear time (all *P*>.15). The day of the week was related to valid wear time, with valid days being less likely to occur on weekend days (odds ratio 0.25, 95% CI 0.17-0.37; *P*<.001).

**Table 1:** Characteristics of the study sample and descriptive statistics of main study variables (participants: N=15; 636 ecological momentary assessment prompts).

Characteristics	Values
Age (years), mean (SD)	13.07 (1.03)
Sex (female), n (%)	10 (67)
Ethnicity (Hispanic), n (%)	6 (40)
Highest maternal education (college and above), n (%)	11 (73)
Weight status (healthy weight), n (%)	10 (67)
BMI percentile, mean (SD)	55.42 (32.05)
ActivPAL SB^a,^^b^ (minutes), mean (SD)	11.88 (4.24)
Screen-based SB (yes), n (%)	257 (40.4)
Negative affect, mean (SD)	0.29 (0.60)
Positive affect, mean (SD)	1.58 (0.97)

^a^ActivPAL-measured SB in the 15-minute window before the ecological momentary assessment prompt.

^b^SB: sedentary behavior.

### Descriptive Statistics

Table 1 presents descriptive statistics of the main study variables. Across all answered EMA prompts, participants reported an average negative affect of 0.29 (SD 0.60) and an average positive affect of 1.58 (SD 0.97). On occasions when screen-based SBs were reported, the mean negative affect was 0.40 (SD 0.76), whereas it was 0.22 (SD 0.44) on occasions when screen-based SBs were not reported. On occasions when screen-based SBs were reported, the mean positive affect was 1.75 (SD 0.98), whereas it was 1.47 (SD 0.94) on occasions when screen-based SBs were not reported.

### SB and Negative Affect

**Figure 1:**
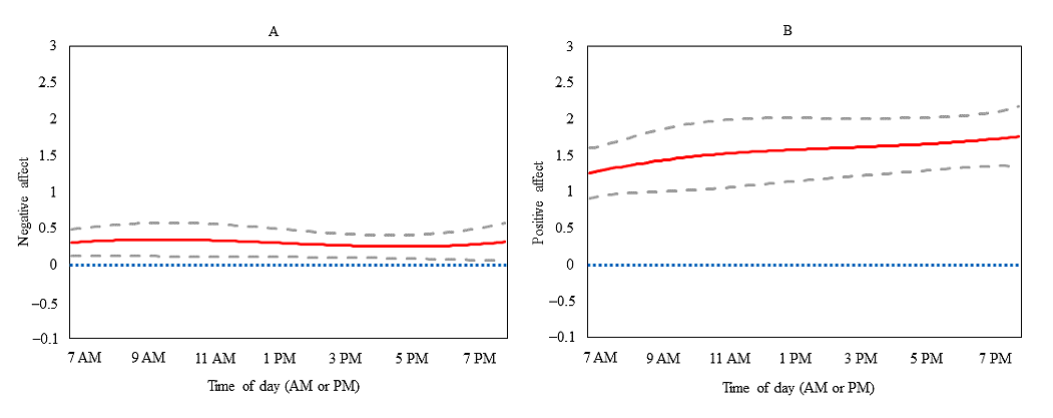
Intercept-only time-varying effect model plots depicting unadjusted average negative affect (panel A) and unadjusted average positive affect (panel B) from 7 AM to 8 PM (N=636). The solid red line represents the point estimate; the dashed gray lines represent the corresponding 95% CI.

**Figure 2:**
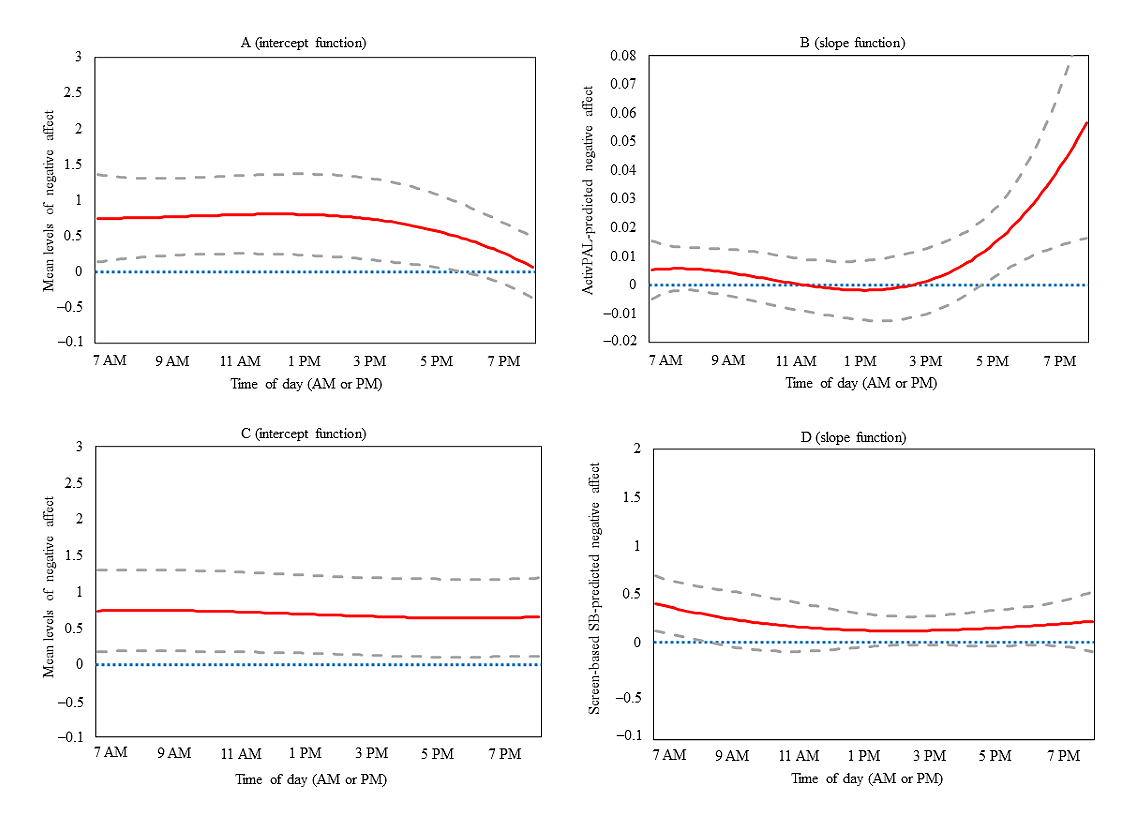
Time-varying effect model plots depicting the intercept and slope functions of the association between SB and concurrent negative affect from 7 AM to 8 PM. The intercept function represents momentary levels of negative affect, adjusted for covariates. The slope functions represent the adjusted estimate of the association (*β*) between SB and concurrent negative affect. Panels A and B present estimates from the activPAL model (N=542). Panels C and D present estimates from the ecological momentary assessment–reported screen-based SB model (N=636). The solid red line represents the point estimate; the dashed gray lines represent the corresponding 95% CI. SB: sedentary behavior.

The intercept-only (unadjusted for covariates) TVEM plot for negative affect is presented in Figure 1 (panel A). The mean level of EMA-reported negative affect remained steady at around 0.30 across the daily EMA-prompting period (7 AM to 8 PM). The highest levels of negative affect were reported just before 10 AM (mean negative affect 0.35, 95% CI 0.12-0.58) and the lowest levels of negative affect were reported at approximately 5 PM (mean negative affect 0.25, 95% CI 0.09-0.41). The intercept functions in Figure 2 (panel A and panel C) indicate that negative affect was 0.06 to 0.81 across the entire day, adjusting for the average levels of all covariates. The slope function in panel B (Figure 2) presents the time-varying acute associations between activPAL-measured SB in the 15-minute window before the EMA prompt and EMA-reported negative affect across the day. SB was unrelated to negative affect until just after 5 PM when SB was directly related to concurrent negative affect until 8 PM (*β* range .01-.06) after adjusting for a priori covariates and environmental context. Panel D (Figure 2) presents the time-varying acute associations between EMA-reported screen-based SB and concurrent negative affect from 7 AM to 8 PM, indicating that there was a significant direct association from 7 AM to just before 9 AM (*β* range .29-.41) after adjusting for a priori covariates, social context, and environmental context. Physical activity and LYCOS Life were not significantly associated with the outcome and were therefore not retained in these models.

### SB and Positive Affect

**Figure 3:**
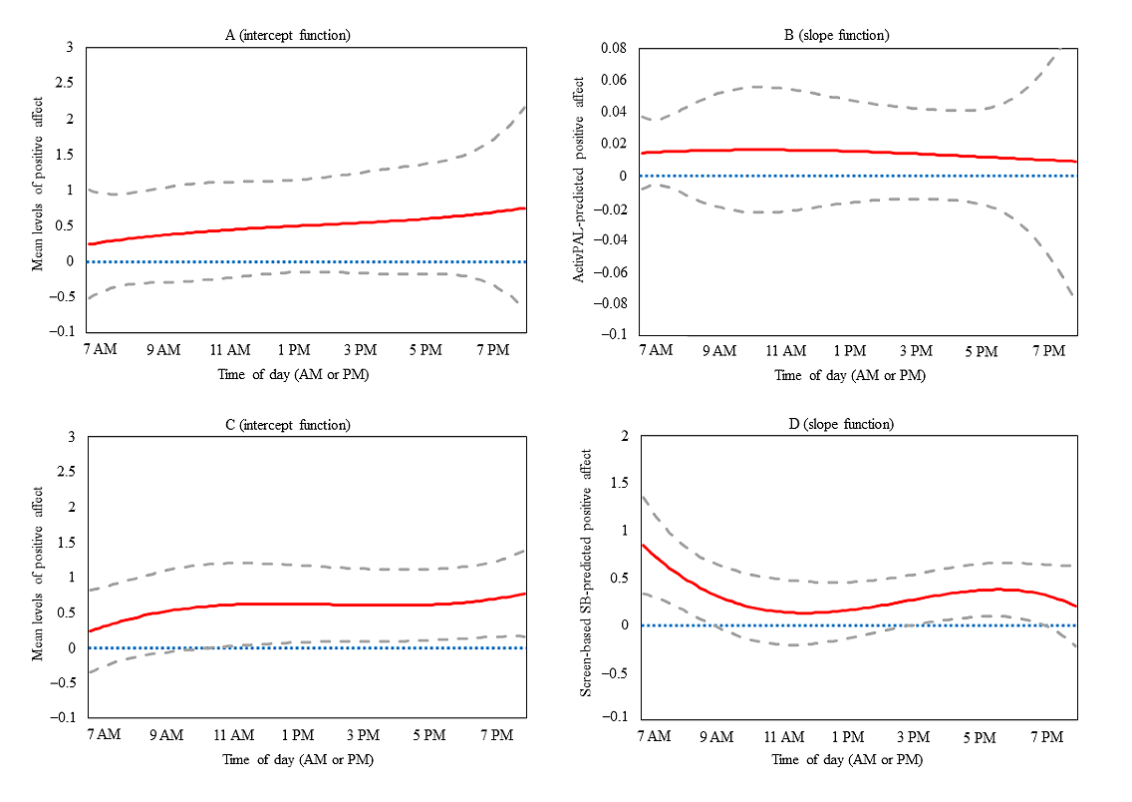
Time-varying effect model plots depicting the intercept and slope functions of the association between SB and concurrent positive affect from 7 AM to 8 PM. The intercept function represents momentary levels of positive affect, adjusted for covariates. The slope functions represent the adjusted estimate of the association (*β*) between SB and concurrent positive affect. Panels A and B present estimates from the activPAL model (N=542). Panels C and D present estimates from the ecological momentary assessment–reported screen-based SB model (N=636). The solid red line represents the point estimate; the dashed gray lines represent the corresponding 95% CI. SB: sedentary behavior.

The intercept-only (unadjusted for covariates) TVEM plot for positive affect is presented in Figure 1 (panel B). The mean level of EMA-reported positive affect slightly increased across the day from 7 AM (mean positive affect 1.25, 95% CI 0.90-1.59) to 8 PM (mean positive affect 1.76, 95% CI 1.35-2.18). The intercept functions in Figure 3 (panel A and panel C) indicate that positive affect was 0.22 to 0.78 across the entire day, adjusting for average levels of all covariates. The slope function (panel B, Figure 3) presents the time-varying acute associations between activPAL-measured sedentary time and positive affect, demonstrating that these associations were nonsignificant across the day (from 7 AM to 8 PM). Panel D (Figure 3) presents the time-varying acute associations between EMA-reported screen-based SB and positive affect across the day; significant direct associations were observed from 7 AM to just after 9 AM (*β* range .35-.88) and from just after 3 PM to just after 7 PM (*β* range .27-.38). Each of the abovementioned models was adjusted for a priori covariates but was not adjusted for additional potential covariates (eg, physical activity, environmental context, social context, and LYCOS Life) as they were not associated with the outcome.

### Sensitivity Analyses

After removing participants with no prior smartphone ownership (2/15, 13% of participants who collectively contributed 95/636, 14.9%, completed EMA prompts), the time-varying associations between activPAL-measured SB, screen-based SB, and concurrent affective states remained comparable with those presented previously. Therefore, providing a smartphone to those who otherwise might not have had regular access to a smartphone did not appear to influence our results, and these participants were retained in the final models. Multimedia Appendix 4 presents the frequency of EMA-reported screen-based SB by the time of day (EMA-prompting window) and day of the week (weekend day vs weekday). The frequency of screen-based SB did not differ by day of the week during any time of day (all chi-square *P*>.05). In addition, all models were rerun using data from weekend days only, and the results remained comparable with the main study findings. Therefore, all models presented data from weekdays and weekend days combined, with the day of the week as a covariate.

## Discussion

### Principal Findings

In this proof-of-concept study, we provided initial evidence to suggest that the associations between SB and concurrently reported affective states may differ by time of day and SB measurement modality. We did so by leveraging the TVEM, device-based activity monitoring, and EMA. We found that device-based SB was associated with more concurrent negative affect, possibly only in the evening. Our findings also indicated that device-based SB may be unrelated to concurrent positive affect across the entire day. Alternatively, EMA-reported screen-based SBs were related to more concurrent negative affect, possibly only in the morning. EMA-reported screen-based SBs also appeared to be related to more concurrent positive affect in the morning and late afternoon. Taken together, our results indicate that there may be critical windows during the day in which specific types of SBs tend to co-occur with affective states. This could have important intervention implications if future confirmatory studies using similar methodologies can replicate these findings.

### Comparison With Prior Work

We demonstrated that the direct association between screen-based SBs and concurrent negative affect may only be significant during morning hours (7 AM to approximately 9 AM). These findings are in line with a previous longitudinal study of adolescents, which found that screen-based SBs were bidirectionally associated with negative affective depressive symptoms but not with other types of depressive symptoms [35]. In contrast, although previous evidence suggests that engagement in screen-based SB, specifically in the evening, is related to negative affect, we did not observe such associations in the evening hours [17]. It is believed that engagement in screen-based SBs later in the day may influence sleep duration or quality, which in turn affects other factors such as mood and executive function [36]. However, our findings may not be consistent with this notion as the most commonly reported screen-based SB in our sample was television viewing (approximately 50% of all EMA prompts when engagement in screen-based SB was reported). Prior cross-sectional and longitudinal research suggests that compared with other forms of screen-based SB, television viewing is not as strongly related to emotional outcomes (and vice versa), perhaps because of its passive nature [37]. In contrast, nighttime engagement in other types of active screen-based SBs, such as computers and mobile phones, may be more strongly related to emotional health than television viewing [17,38,39]. Therefore, further research is needed on the potential for device- and time-specific associations between screen-based SB and negative affect, and the possible roles of sleep duration and quality should be further explored.

We also demonstrated that engagement in screen-based SBs may be related to more concurrent positive affect during the morning and afternoon hours. Depending on the time of day, adolescents may view screen-based SBs as pleasurable activities for coping with stressors [40,41]. Participants in our sample may have engaged in screen-based SBs in the morning hours to relax in preparation for the upcoming day at school (weekdays) or the day of structured organized activities (weekend days). Similarly, our sample may have engaged in screen-based SBs in the afternoon hours to attempt to alleviate stress from academics on weekdays and unwind from overscheduling on weekend days, which can be common during adolescence [42-44]. Longitudinal and bidirectional evidence across 1 year indicates that screen-based SBs are unrelated to positive affect in both directions [35]. Youth may choose behavioral coping strategies (ie, screen-based SBs) to manage or improve mood following a stressor; however, these coping strategies are considered maladaptive because while affective states may acutely improve, emotional and physical health are more likely to worsen long-term [45]. Therefore, the within-day association between engagement in screen-based SBs and higher positive affect may be transitory and likely does not accumulate into longer-term emotional or physical health benefits.

Our analyses of device-based SB in relation to affective states yielded differential findings compared with our analyses of EMA-reported screen-based SBs. Device-based SB was directly related to concurrent negative affect in the evening hours (approximately 5-8 PM) in this study. A previous free-living study of the acute (eg, past 30 minutes) associations between device-based sedentary time and negative affect among youth did not find that within-person deviations from one’s usual sedentary time were related to negative affect [14]. Our use of the TVEM provides insight into a potential source of this inconsistency in the study findings. Other modeling methods typically used for multilevel data (EMA data) are parametric and may impose linearity on conceptual time, whereas associations across time may be nonlinear [46,47]. Therefore, when the strength of the association of interest may nonparametrically differ as a function of time, TVEMs may reveal associations that other common parametric modeling methods may not be able to capture [48]. Given our findings, the acute associations between SB and affective states appear to be nonlinear and nonparametric functions of the time of day. Therefore, future studies should consider taking a TVEM approach to understand the within-day associations between behaviors and affective states. Additional evidence from studies such as ours is needed to identify possible time windows of opportunity (when associations between behavior and affect are strongest) for intervention strategies to be delivered.

The differential findings between screen-based SB and device-based sedentary time also highlight that each is a distinct, yet interrelated, nuance of a larger behavior, broadly referred to as SB. A previous study among youth found that EMA-reported screen-based SBs were highly correlated with device-based sedentary time [49], and ancillary analyses of our sample also support this finding. Together, this suggests that our participants minimally misreported their engagement in screen-based SB via the EMA surveys. Therefore, the differences in associations by SB measurement modality (eg, EMA-reported screen-based SBs vs device-based sedentary time) observed in our study were likely not because of recall biases or errors. Rather, our study supports the notion that the behaviors performed while sedentary may uniquely relate to affective states in addition to objective time spent sitting. Altogether, our findings suggest that future investigations of SB–affective state associations should consider approaches that combine device-based measures of sedentary time and self-reported engagement in screen-based SBs, as they provide complementary behavioral information.

### Strengths and Limitations

The strengths of this study include the real-time, repeated-measures data collection methods that were leveraged, which allowed us to model complex temporal associations and within-day changes in the association between SB and affective states using the TVEM. As EMA and accelerometry are data collection strategies that capture data in a naturalistic setting, this study was ecologically valid. Furthermore, to the best of our knowledge, this was the first study that combined EMA and device-based SB to directly examine how the operationalization of SB may have influenced the strength of the associations at hand.

However, there are also limitations that warrant further discussion. For brevity of our EMA surveys, we only used 5 items from the Positive and Negative Affect Schedule–Child survey to capture affective states. Although this is consistent with previous EMA studies on youth [10,19], future work could consider using more survey items to capture affective experiences. Another limitation of this study is that participants were given a wrist-worn activity monitor (LYCOS Life) to prompt them to walk every 30 minutes during one of the assessment weeks. Although the wrist monitors and EMA surveys were not programmed to coordinate with one another, it is important to note as frequent bouts of walking may be related to improved affective states [50]. Although we attempted to statistically control for the wrist-worn activity monitor by including it as a covariate in our models, this may not have entirely accounted for its impact on our findings. This is because the wrist-worn device could have influenced other unmeasured factors such as motivation, self-regulation, and social desirability [51,52]. Future studies should attempt to address this limitation.

In addition, our EMA-prompting period spanned from 7 AM to 8 PM; therefore, our findings cannot be generalized beyond these times of the day. Similarly, as our EMA-prompting schedule did not ask participants about their SBs and affective states from 8 AM to 3 PM on weekdays (because of school or summer camp schedules), the midday estimates presented were driven by weekend day data. This prompting schedule also did not allow us to stratify the models by weekend days versus weekdays. Future studies should attempt to address this limitation by prompting participants across the entire day on both weekend days and weekdays.

It is also worth noting that the EMA prompt compliance among our sample was slightly below that previously reported among other nonclinical samples of youth [53]. In addition, participants were less likely to complete EMA prompts during the morning hours, perhaps because the EMA-prompting schedule started too early in the day. To gain a better understanding of how missing data may influence the findings, models were rerun after the removal of the participant with the lowest EMA prompt compliance (approximately 33%). These results are comparable with those presented previously. Future studies with larger sample sizes should attempt to gain a better understanding of how missing data may affect study findings by stratifying analyses by participant compliance (ie, in those above vs below the average level of compliance).

The characteristics and size of our sample are also limitations of this study. For example, our participants experienced relatively low and stable levels of negative affect, limiting our ability to detect the effects of SB on negative affective states. Therefore, it is possible that some of our null findings may not be entirely because of a lack of association between SB and negative affect. However, this study warrants future confirmatory studies with larger samples, which would introduce more variability in negative affect. Power analyses for TVEMs are currently an open area of research [54]; therefore, post hoc power calculations were not completed. However, the CIs generated from TVEMs reflect the amount of data contributed at each time interval (CIs are wider on occasions when there are fewer data points) [54], aiding our understanding of the statistical power in this study. Future studies with larger samples (at the EMA prompt and person levels) are warranted as it is possible that this study was statistically underpowered, partially contributing to our null findings at some time points within the day. Studies with larger samples will also allow for the investigation of a more nuanced operationalization of the SB construct (eg, subtypes of screen-based SB) in relation to affective states across the day. A better understanding of how the different subtypes of screen-based SB relate to affective states will aid in the development of tailored intervention strategies targeting the forms of SB that appear to be most important for affective experiences. Finally, because of the time reference specified in the EMA item wording (ie, SB and affective states *right now*), this study only assessed concurrent associations.

### Conclusions

This proof-of-concept study demonstrated that within-day associations between SB and affective states may not be static. Rather, these associations may differ by time of day and measurement method of SB, indicating that there may be critical windows during the day in which specific types of SB can be related to concurrent affective states. Therefore, we provide preliminary evidence to suggest that future confirmatory studies aimed at investigating the SB–affective state relationship should consider the possible time-varying nature of these associations. Future studies should also investigate the underlying variables that help explain these possible time-dependent variations in youth. We also demonstrated that self-reported screen-based SBs and device-based SB are likely distinct constructs that may be uniquely related to affective experiences. Together, we provide a preliminary justification for future investigators to carefully consider the statistical modeling and SB measurement methods they choose to use.

## Appendix

Multimedia Appendix 1 Ecological momentary assessment prompting schedule for this study.

Multimedia Appendix 2 Ecological momentary assessment survey item wording, response options, and formatting for each prompt during the assessment period.

Multimedia Appendix 3 Ecological momentary assessment prompt compliance by participant characteristics (1030 prompts; N=15 participants).

Multimedia Appendix 4 Frequency of ecological momentary assessment–reported screen-based sedentary behavior stratified by time of day and day of week.

## Abbreviations

EMA: ecological momentary assessment
MATCH: Mothers’ and Their Children’s Health
SB: sedentary behavior
TVEM: time-varying effect model
