# Supplementary material for: Time-Varying Associations Between Device-Based and Ecological Momentary Assessment–Reported Sedentary Behaviors and the Concurrent Affective States Among Adolescents: Proof-of-Concept Study
Source: JMIR Form Res. 2022 Jun 10;6(6):e37743. doi: 10.2196/37743 (PMC9233247; doi:10.2196/37743)
Supplement: Multimedia Appendix 1 [file formative_v6i6e37743_app1.docx]

**Multimedia Appendix 1.** Ecological momentary assessment prompting schedule for the current study

| Daily Measurement Schedule | 7am-8am | 9am-10am | 11am-12pm | 1pm-2pm | 3pm-4pm | 5pm-6pm | 7pm-8pm |
| --- | --- | --- | --- | --- | --- | --- | --- |
| Weekdays | X |  |  |  | X | X | X |
| Weekend days | X | X | X | X | X | X | X |
