# Supplementary material for: Time-Varying Associations Between Device-Based and Ecological Momentary Assessment–Reported Sedentary Behaviors and the Concurrent Affective States Among Adolescents: Proof-of-Concept Study
Source: JMIR Form Res. 2022 Jun 10;6(6):e37743. doi: 10.2196/37743 (PMC9233247; doi:10.2196/37743)
Supplement: Multimedia Appendix 2 [file formative_v6i6e37743_app2.docx]

**Multimedia Appendix 2.** Ecological momentary assessment survey item wording, response options, and formatting for each prompt during the assessment period

| Construct | Items | Response Options | Format |
| --- | --- | --- | --- |
| Positive and Negative Affect  (PANAS-C short) | Just before the phone went off, how (HAPPY, JOYFUL, STRESSED, MAD, SAD) were you feeling? | Not at all  A little  Quite a bit  Extremely | Separate screen for each item |
| Sedentary Behavior | Please choose the ONE main sedentary activity you were doing just before the phone went off. | TV/Movies/Videos  Texting  Social Media (Facebook, Snapchat, Instagram, Tumblr, etc.)  Video Games  Computer Use/Tablet Use  Homework/Reading  Hanging Out/Chatting  Art/painting/coloring  Riding in the car/bus  None of these things | Single screen |
| *Covariates* |  |  |  |
| Physical Activity | Please choose the ONE main physical activity you were doing just before the phone went off. | Exercise  Sports  Walking/Biking for Transportation  Active House Chores  None of These Things | Single screen |
| Environmental Context | Were you inside or outside just before the phone went off? | Inside  Outside | Single screen |
| Social Context | Who were you with just before the phone went off? (Choose all that apply) | Mom  Dad  Sister(s) or Brother(s)  Other Family Members (cousins, uncles)  Friend(s)  Classmate(s)  People You Don’t Know  Other  I Was Alone | Single screen |
