# Supplementary material for: Time-Varying Associations Between Device-Based and Ecological Momentary Assessment–Reported Sedentary Behaviors and the Concurrent Affective States Among Adolescents: Proof-of-Concept Study
Source: JMIR Form Res. 2022 Jun 10;6(6):e37743. doi: 10.2196/37743 (PMC9233247; doi:10.2196/37743)
Supplement: Multimedia Appendix 3 [file formative_v6i6e37743_app3.docx]

**Multimedia Appendix 3.** Ecological momentary assessment prompt compliance by participant characteristics (N=1030 prompts; N=15 participants)

|  | **Entire Sample** | **< 13 years old** | **>=13 years old** | **Boys** | **Girls** | **Non-Hispanic** | **Hispanic** | **Maternal Ed. <College** | **Maternal Ed. College +** | **Healthy Weight** | **Overweight/**  **Obese** | **Weekdays** | **Weekend Days** |
| --- | --- | --- | --- | --- | --- | --- | --- | --- | --- | --- | --- | --- | --- |
| Missed Prompts | 394 | 82 | 312 | 122 | 272 | 222 | 172 | 92 | 302 | 292 | 102 | 213 | 181 |
| Answered Prompts | 636 | 118 | 518 | 231 | 405 | 389 | 247 | 189 | 447 | 414 | 222 | 362 | 274 |
| Compliance | 61.74% | 59.00% | 62.41% | 65.44% | 59.82% | 63.67% | 58.95% | 67.26% | 59.68% | 58.64% | 68.52% | 62.96% | 60.22% |
