# Supplementary material for: Time-Varying Associations Between Device-Based and Ecological Momentary Assessment–Reported Sedentary Behaviors and the Concurrent Affective States Among Adolescents: Proof-of-Concept Study
Source: JMIR Form Res. 2022 Jun 10;6(6):e37743. doi: 10.2196/37743 (PMC9233247; doi:10.2196/37743)
Supplement: Multimedia Appendix 4 [file formative_v6i6e37743_app4.docx]

**Multimedia Appendix 4**. Frequency of ecological momentary assessment-reported screen-based SB stratified by time of day and day of week

|  | 7am-8am | 3pm-4pm | 5pm-6pm | 7pm-8pm |
| --- | --- | --- | --- | --- |
| *Weekday* | *13 (16.5%)* | *35 (39.8%)* | *39 (41.5%)* | *50 (49.5%)* |
| *Weekend day* | *7 (24.1%)* | *24 (57.2%)* | *17 (40.5%)* | *20 (43.5%)* |
